# Classification of Rice Yield Using UAV-Based Hyperspectral Imagery and Lodging Feature

**DOI:** 10.34133/2021/9765952

**Published:** 2021-03-30

**Authors:** Jian Wang, Bizhi Wu, Markus V. Kohnen, Daqi Lin, Changcai Yang, Xiaowei Wang, Ailing Qiang, Wei Liu, Jianbin Kang, Hua Li, Jing Shen, Tianhao Yao, Jun Su, Bangyu Li, Lianfeng Gu

**Affiliations:** ^1^Institute of Crop Sciences, Ningxia Academy of Agriculture and Forestry Science, Yinchuan, Ningxia 750105, China; ^2^Basic Forestry and Proteomics Research Center, College of Forestry, Fujian Agriculture and Forestry University, Fuzhou 350002, China; ^3^State Key Laboratory of Marine Environmental Science, Xiamen University, China; ^4^Digital Fujian Institute of Big Data for Agriculture and Forestry, Key Laboratory of Smart Agriculture and Forestry, Fujian Agriculture and Forestry University, Fuzhou 350002, China; ^5^Seed Workstations of the Ningxia Hui Autonomous Region, Yinchuan, Ningxia 750004, China; ^6^Aerospace Information Research Center, Institute of Automation, Chinese Academic Science, Beijing 100190, China

## Abstract

High-yield rice cultivation is an effective way to address the increasing food demand worldwide. Correct classification of high-yield rice is a key step of breeding. However, manual measurements within breeding programs are time consuming and have high cost and low throughput, which limit the application in large-scale field phenotyping. In this study, we developed an accurate large-scale approach and presented the potential usage of hyperspectral data for rice yield measurement using the XGBoost algorithm to speed up the rice breeding process for many breeders. In total, 13 japonica rice lines in regional trials in northern China were divided into different categories according to the manual measurement of yield. Using an Unmanned Aerial Vehicle (UAV) platform equipped with a hyperspectral camera to capture images over multiple time series, a rice yield classification model based on the XGBoost algorithm was proposed. Four comparison experiments were carried out through the intraline test and the interline test considering lodging characteristics at the midmature stage or not. The result revealed that the degree of lodging in the midmature stage was an important feature affecting the classification accuracy of rice. Thus, we developed a low-cost, high-throughput phenotyping and nondestructive method by combining UAV-based hyperspectral measurements and machine learning for estimation of rice yield to improve rice breeding efficiency.

## 1. Introduction

Rice (*Oryza sativa* L.) is one of the major cultivated crops worldwide and serves as a staple and primary food source in various countries. It is sown on 7% arable land, which feeds 21% of the world population [1]. Recently, the growth rate of grain production has slowed down noticeably while the food demand in several parts of the world is increasing. For instance, in China, the demand for rice production is expected to increase by about 20% by 2030 [2]. However, the great demand meets with great challenges such as the reduction of labor population due to the demographic development, decline of arable land quality, shortage of water resources, and the climate change [3]. Thus, increasing the yield per hectare of cropped area is an effective way to solve the food demand.

Breeding rice is an extremely complicated process. High-yield rice is affected by weather and other environmental factors, and in various regions, different cultivars are the highest yielding. Estimation of accurate biomass of crops is important in the selection process of conventional breeding and heavily depends on manual measurements. However, manual measurements of crop biomass are subjective and lack robustness or repeatability and are time consuming for larger areas of farm land [4]. Thus, high-throughput phenotyping systems are rapidly evolving in field condition [5], and remote sensing tools, such as RGB cameras [6, 7] and multispectral, hyperspectral, fluorescence, and thermal sensors [4], are used to facilitate data collection in many breeding programs. With the advancement of drone technology and spectral imager manufacturing technology, UAV-based hyperspectral cameras are becoming more and more widely used in agriculture [8]. One key advantage is the flexibility of employment over large areas without the requirement of prior installed stationary platforms along fields. To date, high-throughput phenotyping technologies are frequently used for plant identification and classification, quantifying pigments [9] and monitoring of biotic stresses [10] as well as assessing both simple and complex plant traits such as plant height, biomass, estimation of biochemical parameters, prediction of flowering time, and grain yield [4, 11, 12]. To this end, various reflectance-derived vegetation indices associated with various plant traits have been established in the last decades as easy-to-employ, noninvasive measures [13]. For instance, the normalized difference vegetation index (NDVI) is a highly used index to predict yield and was successfully applied in rice [14], wheat [15, 16], maize [17], sugarcane [18], and sorghum [19]. However, index-based empirical vegetation models are limited by their poor stability due to dynamic environments [20]. Several algorithms have been reported for estimating biomass, such as random forest (RF) [21], multikernel GBLUP approach [22], and multiple linear regression (MLR) models [23]. At present, identification of high-yield rice varieties based on hyperspectral data is often achieved by predictive regression models [24–26].

Machine learning techniques have been widely applied in corn yield estimation [27], disease forecasting [28], classification of abiotic and biotic stresses [29], and discrimination of rice and weed [30]. In this study, we combined hyperspectral data and machine learning for estimation of rice yield. The classifier on the hyperspectral data was trained by the eXtreme Gradient Boosting (XGBoost) algorithm [31]. Compared with traditional machine learning algorithms, including support vector machine (SVM) and RF, XGBoost presents stronger generalization ability and is less prone to overfitting [31]. In addition, the XGBoost algorithm is also suitable for imbalanced data [32]. Moreover, XGBoost implements the parallelization of the algorithm and supports CUDA to accelerate the computing [33], which shortens the training time. This algorithm allows us to classify a large amount of unknown rice cultivars by yield, thereby screening appropriate rice lines for particular agricultural areas. This easy and portable method will provide a high-throughput and cost-effective approach for large-scale screening of high-yield rice lines.

## 2. Material and Methods

### 2.1. Classification of Rice Cultivars according to Manual Measurement of Yield

**Figure 1:**
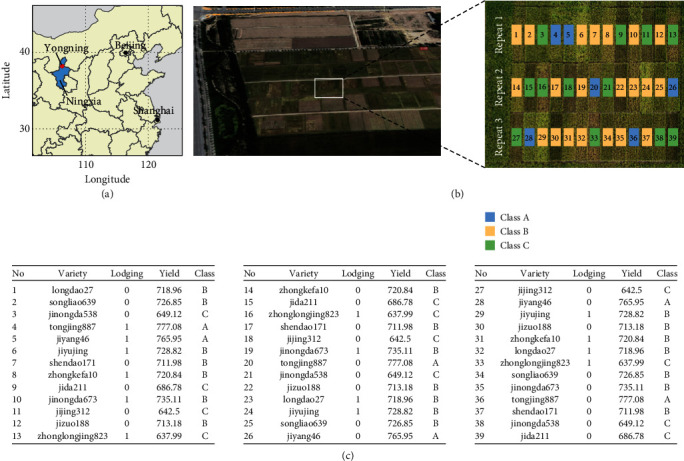
The 13 rice cultivars were divided into different categories according to manual measurements of yield. (a) Open field site for hyperspectral image data collection. (b) Map of the field. In total, 13 rice cultivars were divided into three categories (class A, class B, and class C) according to the manual measurement of yield. (c) Lot IDs and corresponding rice variety, lodging, yield, and category information.

Rice cultivation and hyperspectral image data collection were carried out at the Ningxia Academy of Agricultural Sciences, Yongning County, Ningxia Province, China (38°16′39.36^″^N, 106°14′38.45^″^E) (Figure 1(a)). The average monthly precipitation in Ningxia between June and September is 34.3 mm, and the average temperature in this period varies between 15.9°C and 23.4°C. To minimize the experimental deviation among samples, all treatments, including fertilizers, farming, and spraying, were applied simultaneously to all rice plants. Rice seedlings were transplanted on June 1^st^, 2018, and harvested on September 9^th^, 2018.

In total, 13 early japonica rice lines in late maturity were planted in triplicates in the experimental field (Figure 1(a)). The field was divided into 3 rows with 13 grid cells each. Every row contained a single replicate of each line, and the order was altered among different rows (Figure 1(b)). The rice lines in different grid cells were labeled according to the average amount of manually measured grain yield per mu (Figure 1(b)). According to China's national approval (No:20000011), lines with a yield of more than 750 kg mu^−1^, between 750 and 700 kg mu^−1^, and less than 700 kg mu^−1^ were grouped into class A, class B, and class C, respectively. Class A contained two lines, seven lines were sorted in class B, and four lines belonged in class C. In addition, the presence or absence of a lodging phenotype was documented with 1 or 0, respectively (Figure 1(c)).

### 2.2. UAV-Based Hyperspectral Image Data Collection

**Figure 2:**
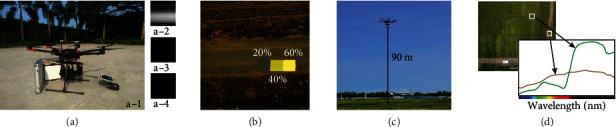
Hyperspectral camera calibration and image acquisition. (a) Calibration of the exposure time with a standard whiteboard in sunlight (a-1) including taking a white image (a-2), dark background image (a-3), and dark background image with increased exposure time (a-4). (b) Reference reflectance panels of 20%, 40%, and 60% reflectivity, respectively. (c) UAV flying at a height of 90 m during image acquisition. (d) Exemplary sample area and corresponding spectral curve of rice and soil.

**Figure 3:**
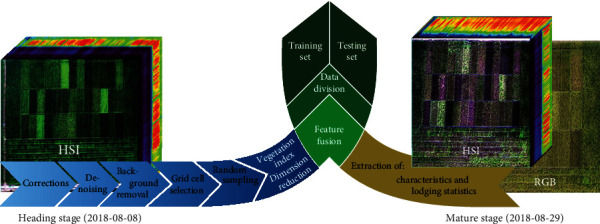
Processing workflow of hyperspectral and RGB images and dataset construction. Raw images from heading stage were adjusted for several types of distortions to obtain corrected data. Reference spectra were extracted, and noise and background were removed. After grid cell and subsequent ROI selection, random pixels were used to calculate vegetation indices. Lodging statistics from mature stage were extracted and combined with the spectral data to provide training and testing set.

**Figure 4:**
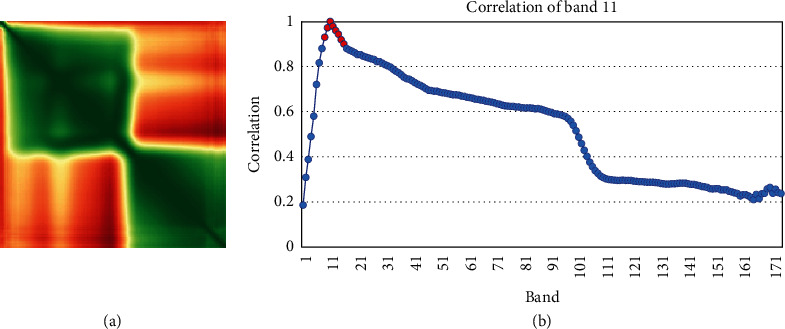
Number of combining consecutive bands with correlation coefficients. (a) Band correlation matrix from one rice field in Wangtai region. (b) Correlation curves between the 11th band and other bands. The correlation from the 8th to 17th bands exceeded 0.9, and the information could be considered redundant.

In this study, a DJI M600 Pro hexacopter was used as the flight platform equipped with a 176-band hyperspectral camera, which was produced by Dualix (GaiaSky-Vis&Nir) (Figure 2(a), a-1). Prior to measurements, the exposure time was calibrated in direct sunlight. One white and two black background images were acquired. For the white image, a diffuse reflectance standard was placed perpendicular in front of the lens (Figure 2(a), a-2). The two dark background images (Figure 2(a), a-3 and a-4) were taken according to the manufactural specifications with closed lens cap and regular or increased exposure time, respectively. The exposure time for the whiteboard and dark background of the hyperspectral camera was 0.9 s and 1.0 s, respectively. For radiation calibration, reference panels of 20%, 40%, and 60% reflectivity were placed next to the field and during image processing used as standards (Figure 2(b)). The resolution of this hyperspectral camera was 960 × 1057 pixels and yielded a 4.5 cm spatial resolution at 90 m flight altitude (Figure 2(c)). The hyperspectral camera is a line sensor which had a wavelength range reaching from 400 to 1000 nm, with a spectral resolution (FWHM) of 3.5 nm and exposure time of 7 s each image. The spectral curve of rice was different from the surrounding soil (Figure 2(d)). Before the data collection, we checked the field to make sure that the rice fields only included rice without other weeds to disturb the hyperspectral data.

We collected hyperspectral data on August 8^th^, 2018, and August 29^th^, 2018, which corresponded to the filling stage and the midmature stage of rice, respectively. Both UAV flights were conducted between 10 AM and 2 PM under sunny and cloudless weather conditions for minimizing the possible influences of shadow. The drone was hovered at a height of 90 m during the image acquisition and controlled by DJ GO utilize GPS (Global Positioning System) service. The exposure time during aerial measurements is 1.0 s. The HSI sensor is a line sensor. The period between two HSI images captured is about 7 s–9 s. During the midmature stage, we documented lodging phenotype in planting areas, since in case of lodging, recorded radiation come from both leaf canopy and stems of rice plants.

### 2.3. Hyperspectral Image Preprocessing

The hyperspectral image preprocessing workflow includes data calibration, noise, and background removal and selection of region of interests (ROIs). Subsequently, we performed random nonrepetitive sampling, calculation of vegetation indices, and data dimension reduction. After combining hyperspectral data, vegetation indices, and lodging characteristics into one dataset, we finally divided our data into a training and a test set (Figure 3). In the following sections, the processing steps will be described in more details. In a first preprocessing step, hyperspectral raw images were adjusted for lens, reflectance, and radiation distortions (Figure 3). For lens correction, we used the lens correction file “LensCorrection960x176.lcf” provided by the camera manufacturer. For reflectance calibration, the preset parameter of the white frame was 34%, and the 3-point curve smoothing method was used. For radiation calibration, we used the gray cloth curve file “reference tile-40%.txt” provided by the camera manufacturer. The corrected data were further noise corrected and the background was removed (Figure 3). Regions of interest containing only rice and no soil as well as ROIs of soil without any rice were selected. A reference spectra library was generated from the average spectra of those ROIs. With this library, original spectra were classified using the spectral angle mapper (SAM) method [34] to remove the background and eliminate soil noise.

Our hyperspectral data contain 176 bands with a higher sensitivity than the analysis requires. We used the average of 8 adjacent bands as the center band because this was the maximum number of combining consecutive bands with correlation coefficients, which exceeded 0.9 (Figure 4). Therefore, we reduced the dimensions to 22 spectra.

### 2.4. Parcel Detection and Random Sampling

To remove the soil background, we first constructed a reference spectral library, which was composed of the average spectral of soil and rice ROIs (region of interest). Then, every pixel of the image was compared with the reference spectral library using the SAM (spectral angle mapper) method to calculate the spectral angle of each pixel by comparing with both soil and rice ROIs. Finally, the soil background was recognized as the pixels which had smaller spectral angle with soil ROIs than rice ROIs. After background removal, the planting area for each line was marked. The edge regions around each rice variety were removed during labeling to exclude the influence of adjacent plants (Figure 3). We used the method of average sampling for each japonica rice line to obtain 300 sample points. In each chosen ROI, 300 points were randomly selected without allowing repetitions as the training set for further modeling. Pixels identified as background and noise were removed during these preprocessing steps, and points with a value of zero (soil background) were excluded from the sampling process.

### 2.5. Calculation of Vegetation and Lodging Indices

Spectral characteristics of vegetation in the visible and near-infrared have been widely used as a qualitative and quantitative measure of vegetation cover, growth vigor, or biomass. In total, 41 different vegetation indices from the literature (Supplementary Table 1) were calculated with our data set (Figure 3). During data collection on August 29^th^, 2018, we documented lodging effects within our test site in several grid cells. We quantified this effect by using the count of repetitions with observed lodging as an index for the line (Figure 1). For example, lines such as “jida211” without any lodging were assigned a 0, while “zhonglongjing823” with lodging in all three replicates was assigned a 3. The lodging index was added as a feature to the sampled data during the feature expansion process (Figure 3). The effect of lodging on the classification was finally assessed by integrating or excluding the lodging index during the training process.

### 2.6. Data Fusion and Intraline/Interline Testing

**Figure 5:**
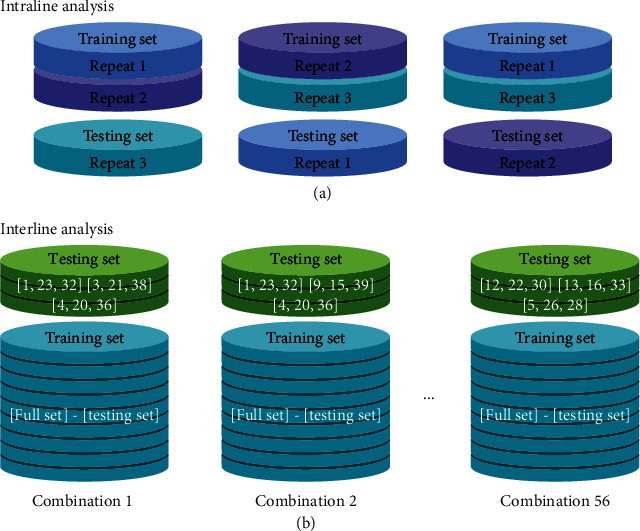
Sample combinations for model establishment. Schematic representation of replicate and variety permutation for intraline (a) and interline (b) analyses, respectively.

The preprocessed spectral data, vegetation indices, and lodging characteristic data were combined (Figure 3) and labeled according to the rice lines. We performed two analyses, an intraline and an interline test. For the intraline analysis, we pooled from each line two randomly chosen repetitions as training data and considered the third repetition as test data (Figure 5(a)). For interline testing, one line from each class was selected and designated as the test sample. The remaining 10 lines were used as training samples. In total, there were 2 (class A) × 7 (class B) × 4 (class C) = 56 permutations between classes (Figure 5(b)). In order to reduce the test error and increase the feasibility of the verification method, all 56 training and test sets were subsequently analyzed.

### 2.7. XGBoost Algorithm Using Grid Search Method

The boosting algorithm refers to a kind of algorithm that implements complex models by combining multiple simple models. The advantage of the boosting strategy is that multiple weak learners can form a high-accuracy model. Gradient boosting is an improved version of the boosting algorithm. The main idea is that each model is based on the gradient descent direction of the loss function of the last established model. In order to continuously improve the model during the optimization process, gradient boosting causes the loss function to fall in the direction of the gradient. XGBoost [31] was developed on the basis of the gradient boosting decision tree. The loss function for every base learner in XGBoost was approximated by the Taylor series. In particular, using the 2nd derivative of the series can efficiently direct the gradient and reduce the load to calculate the loss for potential base learners. The objective function of the XGBoost algorithm is

(1) $$\begin{array}{c} L\left(\phi \right)=\underset{i}{\sum }l\left({\hat{y}}_{i},y_{i}\right)+\underset{k}{\sum }\Omega \left(f_{k}\right),\Omega \left(f\right)=\gamma T+\frac{1}{2}\lambda {\left\|w\right\|}^{2}, \end{array}$$

where $l\left({\hat{y}}_{i},y_{i}\right)$ is the training loss, *Ω*(*f*_*k*_) refers to the complexity of trees and *f*_*k*_ is a regression tree, *T* is the number of leafs in the tree, and *w* represents the score of the regression tree node. In addition, the XGBoost algorithm prevents overfitting and uses random forests to sample data columns [31]. The XGBoost algorithm supports parallel computing, imputes missing values, and learns in an iterative process how to build a base classifier. We determined the optimal parameters of the XGBoost algorithm by the grid search method. In the comparative test with or without lodging characteristics, the training of the model was based on the same parameter space to ensure that the comparison test was not affected by other factors.

## 3. Results

### 3.1. Evaluation of the Hyperspectral Measurements

**Figure 6:**
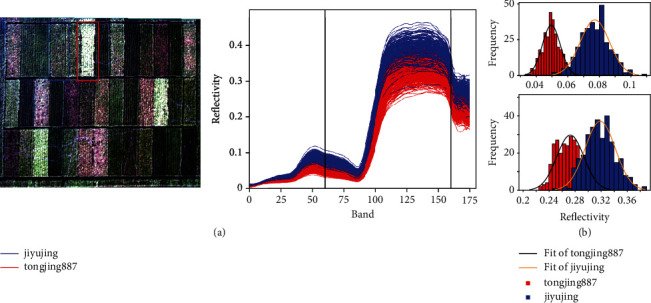
Evaluation of the hyperspectral measurements within and between lines. (a) Spectral curve comparison of “jiyujing” and “tongjing887.” (b) Frequency histogram and fitting curve of “jiyujing” and “tongjing887” in bands 60 (top) and 160 (bottom) and fitted curve.

The spectral reflectance in the experimental field was affected by planting, weather, and other environmental conditions as well as uneven growth, which might lead to minor variations in the spectral curves obtained within a single grid cell. To illustrate the spread between the spectra of individual pixels within and between single replicates, we analyzed hyperspectral curves of 300 randomly selected points of the lines “jiyujing” and “tongjing887” (Figure 6(a)). The procedure's distance [35] of the average spectral curves of “jiyujing” and “tongjing887” is 0, which suggested that the hyperspectral curves from both lines had a similar shape. We selected the 60th and 160th bands of “jiyujing” and “tongjing887” and constructed frequency histograms (Figure 6(b)). The spectral data of the bands followed the normal distribution, and Gaussian curves could be fitted with high accuracy (Shapiro-Wilk tests: *p* > 0.05) to any band of various rice lines. As the spectra of different rice lines overlapped, several sample points were selected for a high confidence class prediction. Using only the 39 mean spectra of grid cells was not feasible, since this would have summarized our data set with too few values for use in a machine learning approach.

**Figure 7:**
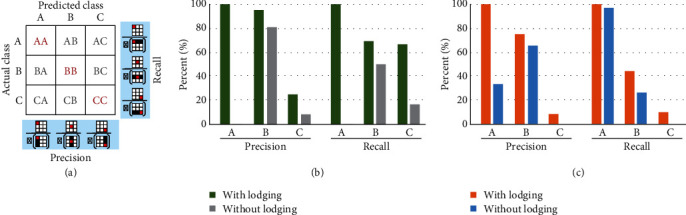
Model evaluation for various analysis strategies. (a) Schematic representation of computation formulas for single class precision and recall rate based on the confusion matrix for classes A, B, and C. (b, c) Average precision and recall rate with and without integration of lodging information for the (b) intraline and (c) interline analyses.

In this study, we conducted a total of four experiments, which included interline tests with (Supplementary Table 2) or without (Supplementary Table 3) lodging characteristics, and intraline tests with (Supplementary Table 4) or without (Supplementary Table 5) lodging characteristics. Each rice line was assigned with an ID (Supplementary Table 6) and compared with all experimental results (Supplementary Table 7). We divide the rice into three categories (class A, class B, and class C) according to the yield. To evaluate the model, we constructed 3 × 3 confusion matrices for predicted results and analyzed the precision and recall rate (Figure 7). Precision and recall rate was calculated as previously described [36]. The single-class precision is the proportion of true positives among all lines predicted to be a member of this class, and the recall rate or sensitivity is the proportion of true positives over the total amount of true class members. We used the single-class precision and recall rate to measure the performance of classifiers since most breeders are more interested in the classification accuracy of the high-yield class A.

### 3.2. Yield Prediction between Repetitions

We first performed an intraline test to ascertain the predictive power of using spectral information to train our classifier (Supplementary Table 5). For this intuitive analysis, we classified our dataset into training and test set according to different repetitions. With precision rates of 0%, 80.95%, and 8.33% for classes A, B, and C, respectively, no feasible model could be built. We therefore incorporated lodging information into our training process. As a result, the newly trained model effectively identifies rice lines of class A, with an accuracy and recall rate of 100% in both cases. The average accuracy and recall rate for class B was 95.24% and 69.26%, respectively.

### 3.3. Yield Prediction of New Rice Cultivars

For breeders, the classification of new rice cultivars or hybrids is of particular interest. This is best reflected by an experimental setup in which entire rice lines are sorted into training or test set. We therefore performed interline analyses building our classifier on 10 cultivars and testing the prediction on the remaining three. When training the predictive model without lodging information, the prediction of class A was consistently low at 33.33% with a very high recall rate for almost all permutations. The predictions of class B varied highly, and in the case of class C, it was not possible to reach any meaningful result.

When integrating lodging information as a feature in the training process, the accuracy and recall rate of class A both increased to 100% (Supplementary Table 2). The average accuracy and the recall rate for class B were 75% and 44.11%, respectively, and in the case of class C, 8.33% and 9.73%, respectively (Supplementary Table 2). During the data analysis, we found that characteristics of class B and class C were relatively close in lodging characteristics and vegetation indices. Thus, the characteristics of class B and class C were relatively close and difficult to distinguish, which clearly affected the accuracy of class C. We consider the accuracy and recall rate for class A as the major factor during the screening process for breeding, since it allows identifying lines with higher yield.

### 3.4. Yield Prediction Only Using Lodging Feature

We further conducted an experiment, only using lodging features to predict the yield. Samples from repeat 1/2 and 3 were used as the training set and testing set, respectively. In total, six different methods were applied for yield based on only lodging feature. The accuracy for LDA (linear discriminant analysis), SVM (support vector machine) with Gaussian kernel, AdaBoost, random forest, and XGBoost was 53.8%, 68.93%, 68.64%, 68.79%, and 69.23%, respectively. Among the above models, XGBoost has the highest accuracy (69.32%), which was still lower than the model that was developed by using combined both HS imaging and lodging features in this study.

### 3.5. Yield Automatic Recognition of Rice Lodging

**Figure 8:**
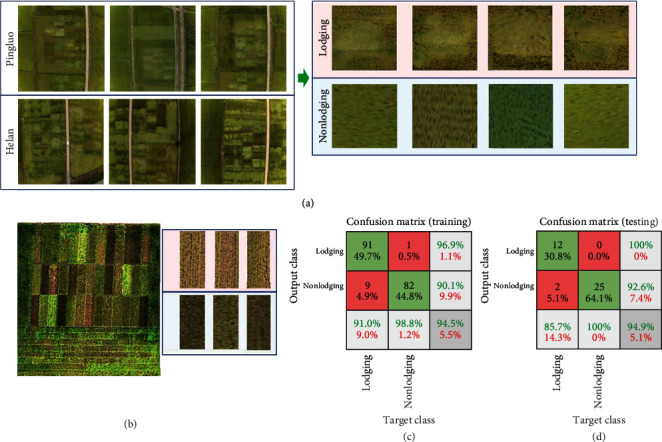
Automatic recognition of rice lodging using fine-tuning technology. (a) The training data for lodging detection model, which included 100 and 83 images for lodging and nonlodging, respectively. (b) Pseudocolor images of hyperspectral data (bands 77, 50, and 18) from 39 experimental fields. The ROIs were intercepted according to planting area to establish testing data set, which included 14 and 25 lodging and nonlodging, respectively. (c) Confusion matrix of the model on the training data. (d) Confusion matrix of the model on the testing data.

Since lodging labels were significant for better estimation of yield, we tested if accurate estimation could be possible without lodging labels by automation of lodging phenotyping. In order to get enough number of samples for training, we collected 100 lodging images and 83 nonlodging images using an RGB camera, which was cheaper than that of the hyperspectral camera during data collection. These images were transformed into unified sizes of 224∗224. Then, lodging ROIs and the nonlodging ROIs were cut out from the aerial images as training data. Finally, we trained a lodging recognition model using fine-tuning technology, image augmentation, and ResNet50 pretraining weights on ImageNet to get an Adam optimizer. The batch size of the model, learning rate, epoch, and iteration was 8, 0.0001, 30, and 600, respectively. We used the trained model to predict lodging in 39 experimental fields for 13 varieties in triplicates (Figure 8). We found that only two lodging cells were predicted as nonlodging cells. Thus, the accuracy of lodging recognition was 94.87%, which suggested that deep learning technology was possible for automatic recognition of rice lodging.

## 4. Discussion

### 4.1. Comparison of Methods for Dimension Reduction

In this study, 13 rice lines at two different growth stages were selected for data collection. By means of the recorded light spectra we were able to distinguish a high-yield from less profitable rice lines. Accuracy and recall are two important reference indicators, which summarizes how many selected items are true positives and how many of the relevant items were successfully identified. Our model has practical application value since it correctly identified rice cultivars with high yield while no middle- and low-yield rice were misclassified as high-yield rice.

In this study, we compared two distinct techniques of data dimension reduction: PCA and mean spectra. For PCA technique, data from 39 plots (300 samples/plot) in spectral space (176 bands) were combined for further dimension reduction by using PCA. In total, 22 components were used for inference. After reducing the dimension of the data and combining both vegetation index and lodging index, we found that when training models using the XGBoost algorithm, the band average method generally led to better classification results than the PCA approach. Although the mean method is effective, it may lose some information. According to our previous study, an autoencoder can perform nonlinear transform and fetch spatial information from HS images [37]. This means an autoencoder is able to acquire higher dimensional features. As the hyperspectral range has rich spectral information and image features, deep autoencoder technology [38] may be effective to compress the hyperspectral data to remove the noise and redundant information.

### 4.2. Comparison of Classification Method and Regression Algorithms

**Figure 9:**
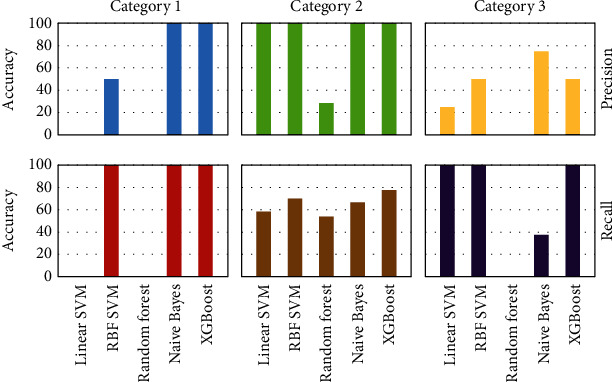
The results of interval tests on five classifiers including linear SVM, RBF SVM, random forest, naive Bayes, and XGBoost.

We used the classification method to screen high-yield rice. Using an appropriate method is crucial to the whole pipeline. In this study, five machine learning algorithms including SVM with linear kernel, SVM with RBF kernel, RF, naive Bayes, and XGBoost were performed and compared. The test results showed that the performance of XGBoost was better than that of the other four methods due to the highest prediction accuracy (Figure 9); thus, we finally adopted XGBoost instead of other methods in the research.

**Figure 10:**
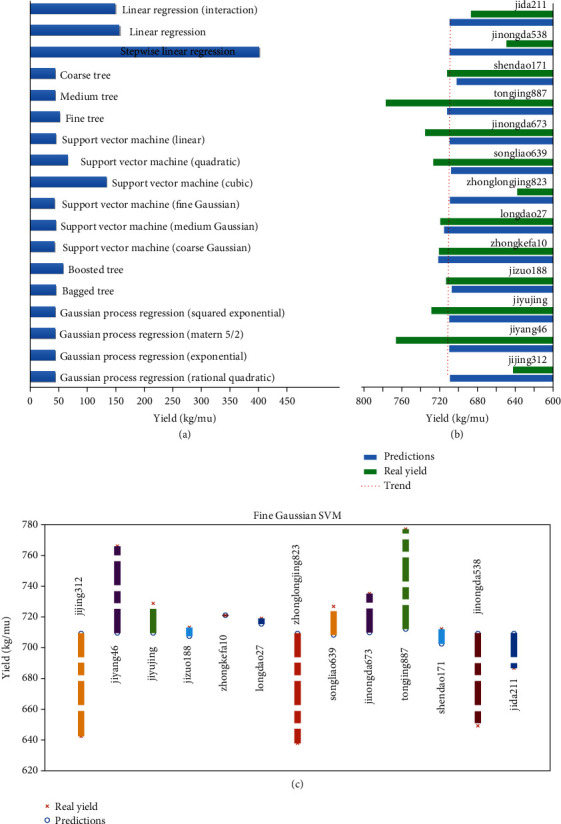
The performance graph on the testing data using trained models. (a) Multiple regression algorithms from MATLAB regression learning toolbox, which showed that fine Gaussian SVM kernel has the smallest RMSE among those regressions. (b) The curve of predicted and real yield using fine Gaussian SVM (c). Residuals of predicted and real yield using fine Gaussian SVM. *x*-axis and *y*-axis presented 13 varieties and yield, respectively.

In addition to classification algorithms, direct prediction of yield using regression analysis was also a possible choice. In this study, we tried the regression method to directly predict the yield instead of yield classification. In brief, we used two repetitions as training sets and the remaining data for validation. We used 19 regression algorithms including linear regression, decision tree regression, SVM (support vector machine), ensemble learning method, and Gaussian process regression. The performance graph on the testing data using trained models showed large deviation between predicted yield data and actual yield data (Figure 10). The maximum deviation reached 72 while the error rate was about 10%. Furthermore, the predicted results fluctuated in the range of 700 kg/mu. After sorting the predicted yields, the order was far different from the real yield. According to a previous study, we found that rice classification according to yield is valuable [39]. Therefore, we did not use regression analysis to directly predict the yield and focused on the classification accuracy of different classes in this study.

### 4.3. Lodging Feature

**Figure 11:**
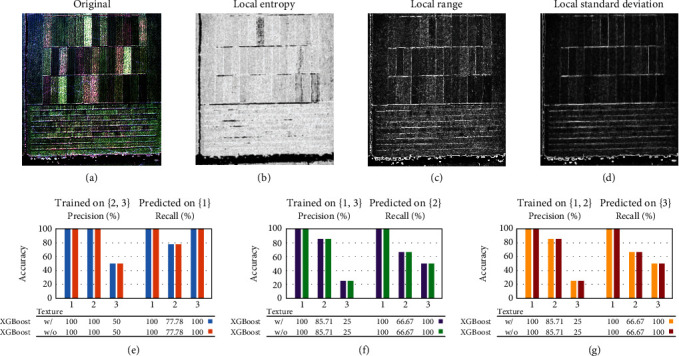
The texture parameters and morphological parameters of the model. (a) Original image. (b) The feature of local entropy yield feature array with each pixel contains the entropy value of the 9-by-9 neighborhood around the corresponding pixel in the input image. (c) The feature of local range contains the range value of the 3-by-3 neighborhood around the corresponding pixel in the input image. (d) The feature of local standard deviation, which is the standard deviation of the 3-by-3 neighborhood around the corresponding input pixel. (e) Comparing the results using the second and third repetitions as the training set and the first repetition as the test set. (f) Comparing the results using the first and third repetitions as the training set and the second repetition as the test set. (g) Comparing the results using the 1st and 2nd repeats as the training set and the 3rd repeat as the test set.

Lodging of crops is a critical factor, which can lead to substantial reduction of yield. Firstly, we trained the predictive model by solely using the lodging features, which has relatively lower model accuracy (up to 69.32%) than that trained by using both HS data and lodging features. Moreover, our comparison between including and excluding lodging information when training the predictive model demonstrated how great the impact of lodging is on the accuracy. To further explore the role of lodging characteristics in the yield classification model, we counted lodging events (Supplemental Table 3). For instance, the two lines of class A, “tongjing887” and “jiyang46,” had a lodging index of 1 indicating mild lodging at midmaturity. Nevertheless, those two japonica lines were full of tassels suggesting that the filling of rice grains in later stages was not impaired. Lines without lodging might indicate a reduced number or length of ears. The precision accuracy of class A was 100%. We suspect that the successful prediction of a particular class correlates with the amount of lodging in the same class. Unfortunately, our dataset contains only one rice category with a lodging index of 1, and further studies are required to test this hypothesis. In addition, since lodging was an important factor for identifying high-yielding rice, the texture parameters and morphological parameters were also worth exploring. In this study, we also used three different texture features, which included local range, local standard deviation, and local entropy (convert RGB image to grayscale image) to improve the yield prediction (Figure 11). Although these texture features including local range, local standard deviation, and local entropy (convert RGB image to grayscale image) have been added in this article, the accuracy of the classification has not been improved. However, there may be other texture or morphological characteristics [40–42] which can heavily affect the prediction of high-yield rice. Considering various factors affecting rice growth, the breeder may dig out more agronomic characteristics related to rice yield in future.

It can be found from the experiments in this article that lodging was an important feature. In this study, we collected spectral and RGB data to assist the breeder to determine the degree of lodging of rice from the mature stage, in which the lodging characteristics were relatively stable. We used the numbers 0 to 3 to indicate the degree of lodging, which were assigned based on the assessment of the rice breeder. Thus, the shortcomings of this classification might lie in the subjective assessment. As the consequence, it would be more objective if the degree of lodging could be represented by some digital features. According to previous works [43, 44], we initially employed a distinct method to reflect the lodging characteristics before we manually evaluated and ranked the lodging degree in this study.

**Figure 12:**
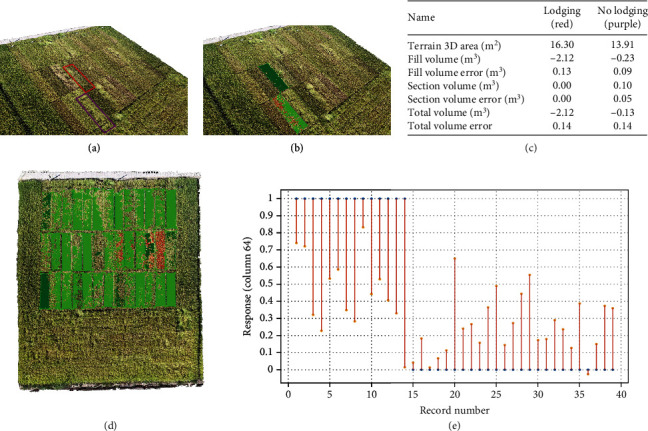
Correlation between features and lodging degree. (a) Point cloud data presented the lodging area and the area without lodging with red and purple, respectively. (b) The tangent plane of the plot. (c) The terrain 3D area (m^2^), fill volume (m^3^), fill volume error (m^3^), cut volume (m^3^), cut volume error (m^3^), total volume (m^3^), and total volume error (m^3^) for the red and the purple area. (d) Volume estimation for all plots. (e) The error of the lodging index generated by the 6 factors and the true label.

We assumed that the corresponding fill volume in rice with lodging characteristics would be relatively large. Therefore, we measured the terrain 3D area (m^2^), fill volume (m^3^), fill volume error (m^3^), cut volume (m^3^), cut volume error (m^3^), total volume (m^3^), and total volume error (m^3^). Then, regression analysis was applied using these seven parameters as independent variables and the rice lodging label identified by breeders in the field as dependent variables. However, the correlation between these features and the lodging degree was too low (Figure 12), which suggested that there will be no suitable threshold to separate lodging and nonlodging characteristics. Therefore, in this study, we directly used the lodging index, which was manually evaluated and ranked. Further research will be needed to find a more appropriate method for measuring digital features to objectively reflect the lodging degree.

Although this study revealed that the lodging characteristics were an important indicator for predicting yield, it would be interesting to know in which growth stage the most accurate prediction can be archived. A previous study shows that a high or low fertilizer level presents different NDVI mean values [45]. Moreover, lodging can also be caused by the growth environment, such as severe storms and rains [46, 47]. In our experiment, lodging occurred at different stages of maturity for various rice lines and to different degrees, suggesting that precise yield prediction at early time points depends on growth conditions (such as fertilizer level or severe growth environment) and potentially vary between lines. The index that we established was based on the detection of lodging in both field and drone images of the midmature stage. Further experiments are required to clarify whether the lodging characteristics in other periods have a similar effect on yield prediction.

### 4.4. Future Direction

UAV-derived high resolution normalized difference vegetation index (NDVI) values at the early reproductive and late ripening stages were most closely related with rice yield using a multispectral sensor system [45]. According to our result, we found that lodging information was one of key factor for yield prediction. However, traditional manual measurement of lodging information is time-consuming. Several previous studies have focused on the lodging characteristics measurement using UAV. For example, a deep learning full-convolution network architecture (UNet) model has been trained using common digital and multispectral images based on a drone platform to determine rice lodging information [48]. In addition, color feature (ExG value), four texture features (coarseness, contrast, line likeness, and directionality), and temperature feature values (mean temperature of every single pixel) are extracted from visible and thermal infrared images for identifying rice lodging areas basing on a support vector machine algorithm [49]. They revealed that high accuracy can be obtained by combining together several features [49]. The digital surface model (DSM), eight texture measures (mean, variance, ASM, entropy, contrast, correlation, dissimilarity, and homogeneity), and the SFP (single feature probability) value are valuable for rice lodging classification based on spatial and spectral hybrid image classification technology [43]. In addition to common digital and spectral images, UAV-LiDAR data from a RIEGL VUX-1UAV sensor was used to generate point cloud data for measuring the height of lodged maize [50]. The canopy height model (CHM) is calculated to obtain the canopy height of maize by subtracting the digital elevation model (DEM) from the digital terrain model (DSM), which is extracted from point cloud data [50]. This study reveals that CHM based on UAV-LiDAR provides accurate measurement of height for lodged maize [50]. From these studies, the trend for classification of rice lodging will be a different combination of sensor, such as multispectral, thermal infrared cameras, and LiDAR sensor system. In particular, more features including color, texture, and temperature will be involved to assist with the assessment of rice lodging. Finally, in addition to the support vector machine and UNet model, multiple algorithms will be applied in lodging characteristics measurement in the future.

Those data were collected from a single location. Geographical location, temperature, moisture, and duration of sunshine might ultimately alter hyperspectral data [51]. Thus, it will be necessary to collect hyperspectral data from multiple locations to ensure the repeatability of the model in different regions. It is possible that hyperspectral recordings of various lines show different degrees of similarities during the growth phase. To identify the optimal growth stage for yield prediction with hyperspectral sensors, it would be interesting to continuously screen during the entire growth period. This could also reveal potential agronomic and phenotypic characteristics, which determine rice yield.

## Data Availability

All data generated or analyzed during this study are included in this published article and its supplementary information files.

## Supplementary Materials

Supplementary 1 Supplementary Table 1: list of calculated vegetation indices.

Supplementary 2 Supplementary Table 2: interline tests with lodging characteristics.

Supplementary 3 Supplementary Table 3: interline tests without lodging characteristics.

Supplementary 4 Supplementary Table 4: intraline tests with lodging characteristics.

Supplementary 5 Supplementary Table 5: intraline tests without lodging characteristics.

Supplementary 6 Supplementary Table 6: lodging index, rice lines, and number.

Supplementary 7 Supplementary Table 7: comparison of experimental results.
